# A post-marketing pharmacovigilance study of triazole antifungals: adverse event data mining and analysis based on the FDA adverse event reporting system database

**DOI:** 10.3389/fphar.2025.1462510

**Published:** 2025-01-23

**Authors:** Yalan Tian, Min Jin, Hong Ning

**Affiliations:** Department of Pharmacy, Mianyang Central Hospital, School of Medicine, University of Electronic Science and Technology of China, Mianyang, China

**Keywords:** voriconazole, posaconazole, isavuconazole, adverse drug events, signal mining, rational drug use

## Abstract

**Background:**

To explore and analyze post-marketing adverse drug event (ADE) signals for voriconazole, posaconazole, and isavuconazole, and to compare the safety differences among the three drugs, aiming to provide insights for rational clinical use.

**Methods:**

Using the Open Vigil 2.1 online tool, extract adverse drug event (ADE) report data for voriconazole, posaconazole, and isavuconazole from the U.S. Food and Drug Administration’s Adverse Event Reporting System (FAERS) database from the time the drugs were marketed up to the third quarter of 2023. Employ the Reporting Odds Ratio (ROR) and Proportional Reporting Ratio (PRR) methods for data mining. Filter out ADE signals detected by both the ROR and PRR methods, and categorize these ADE signals by System Organ Class (SOC) according to the Medical Dictionary for Regulatory Activities (MedDRA 26.0).

**Results:**

A total of 8,898 ADE reports with voriconazole as the primary suspect drug were retrieved, 1,948 for posaconazole, and 944 for isavuconazole. From the basic analysis of the adverse event reports, male patients (50.31%) outnumber female patients (32.11%). In terms of age, the majority of patients are over 45 years old (52.72%). The reports primarily come from the United States, Japan, France, China, and other countries. A total of 607 ADE signals were identified, with 402 for voriconazole, 159 for posaconazole, and 46 for isavuconazole. Voriconazole ADEs primarily involved the following SOCs: Investigations (9.45%), Eye Disorders (8.46%), and Nervous System Disorders (7.21%); Posaconazole ADEs primarily involved the following SOCs: Investigations (13.84%), General Disorders and Administration Site Conditions (11.95%), and Nervous System Disorders (6.29%); Isavuconazole ADEs primarily involved the following SOCs: General Disorders and Administration Site Conditions (15.22%), Hepatobiliary Disorders (10.87%), and Blood and Lymphatic System Disorders (10.87%).

**Conclusion:**

Voriconazole, posaconazole, and isavuconazole all potentially pose safety risks related to hepatobiliary disorders and cardiac disorders. Additionally, voriconazole carries a higher safety risk for eye disorders and nervous system disorders. Newly discovered ADE signals not mentioned in the drug package inserts include voriconazole-induced rhabdomyolysis, posaconazole-induced peripheral neuropathy, and isavuconazole-induced visual impairment and mental confusion. These findings are significant for guiding rational clinical use of these medications.

## 1 Introduction

Invasive fungal disease (IFD) is a major cause of mortality in patients with compromised immune function, such as those with hematological malignancies, hematopoietic stem cell transplants, solid organ transplants, or those using biologics. The most common pathogens are *Candida* and *Aspergillus* species. Triazole antifungal agents are the primary drugs recommended for the prevention or treatment of IFD (Husain and Camargo, 2019). Voriconazole is a second-generation triazole antifungal agent. In recent years, new second-generation triazole antifungal agents, posaconazole and isavuconazole, have been increasingly used in clinical practice due to their broad-spectrum activity, good tolerability, and favorable safety profiles (Jenks et al., 2019). The U.S. Food and Drug Administration (FDA) established the Adverse Event Reporting System (FAERS), which is one of the most comprehensive adverse drug event databases in the world (Pitts, 2015; Kass-Hout et al., 2016). This study aims to mine and analyze real-world clinical data from the FAERS database to explore the characteristics of adverse drug events associated with voriconazole, posaconazole, and isavuconazole. By conducting a preliminary evaluation of the post-marketing safety of these three drugs, the study seeks to provide insights for rational clinical use.

## 2 Materials and methods

### 2.1 Data source and processing

The data for this study were sourced from the FAERS database, collecting reports for three drugs from their market introduction up to the third quarter of 2023. Using the web-based pharmacovigilance analysis tool OpenVigil 2.1, which accesses FAERS data via the OpenFDA online interface (Böhm et al., 2016), we extracted ADE (adverse drug event) reports for the generic names “voriconazole” 、“posaconazole” and “isavuconazole” where the drug role is specified as “Primary Suspect Drug (PS)”. The collected information includes patient age and gender, dosage and administration route, adverse event name, reporter’s profession, and reporting country.

### 2.2 Signal mining and classification

We employed both the Reporting Odds Ratio (ROR) and the Proportional Reporting Ratio (PRR) methods for ADE signal detection. Simultaneous use of ROR and PRR aims to mitigate bias introduced by the selection of control groups. Based on the two-by-two contingency table, as shown in Table 1, one ADE positive signal requires meeting the following conditions simultaneously: report count (a) ≥3; lower limit of the 95% confidence intervals (CIs) of ROR >1; and PRR >2. Higher ROR and PRR values indicate stronger ADE signal intensity, indicating a greater association between the drug and ADEs (Lao et al., 2021). And utilized the Medical Dictionary for Regulatory Activities (MedDRA version 26.0) to systematically classify the ADE signals obtained through mining. This involved standardizing the coding of System Organ Class (SOC) and Preferred Term (PT).

**TABLE 1:** Two-by-two contingency table for the disproportionality analysis.

	Target AEs	Other AEs
Target drugs	a	b
Other drugs	c	d

ROR=ad/bc $95\%\text{CI}=e^{\ln\left(\text{ROR}\right)\pm 1.96\sqrt{\left(\frac{1}{a}+\frac{1}{b}+\frac{1}{c}+\frac{1}{d}\right)}}$ ; $\text{PRR}=a\left(c+d\right)/\left[c\left(a+b\right)\right]$ $95\%\text{CI}=e^{\ln\left(\text{PRR}\right)\pm 1.96\sqrt{\left(\frac{1}{a}+\frac{1}{a+b}+\frac{1}{c}+\frac{1}{c+d}\right)}}$

AEs: adverse events; a: the number of reports containing both the suspect drug and the suspect ADE; b: the number of reports containing the suspect drug with other ADEs (except the event of interest); c: the number of reports containing the suspect ADE, with other medications (except the drug of interest); d: the number of reports containing other medications and other ADEs.

## 3 Results

### 3.1 Basic characteristics of ADE reports

From the FAERS database, 8,898 ADE reports with voriconazole as the primary suspect drug were retrieved, 1,948 for posaconazole, and 944 for isavuconazole. Among these, male patients (50.31%) outnumbered female patients (32.11%), with the majority being over 45 years old (52.72%). The most reports came from the United States, Japan, France, and China, as shown in Table 2.

**TABLE 2:** Basic information on ADEs for voriconazole, posaconazole, and isavuconazole.

Characteristics	Voriconazole		Posaconazole		Isavuconazole	
	Case number, n	Case proportion, %	Case number, n	Case proportion, %	Case number, n	Case proportion, %
Gender						
Female	2,857	32.11	640	32.85	296	31.36
male	4,477	50.31	914	46.92	553	58.58
unknown	1,564	17.58	394	20.23	95	10.06
Age						
<18	586	6.59	152	7.80	38	4.03
18–44	986	11.08	278	14.27	117	12.39
45–64	2,067	23.23	437	22.43	315	33.37
≥65	2,624	29.49	312	16.02	222	23.52
unknown	2,635	29.61	769	39.48	252	26.69
Reporter country						
1	USA 3904	43.88	USA 1052	54.00	USA 416	44.07
2	JPN 968	10.88	FR 167	8.57	CN 134	14.19
3	FR 697	7.83	UK 98	5.03	ES 81	8.58
4	CN 640	7.19	DE 89	4.57	UK 71	7.52
5	UK 349	3.92	CN 64	3.29	FR 66	6.99

### 3.2 Top 20 ADE signals by frequency

A total of 607 ADE signals were mined by the ROR and the PRR method, with 402 related to voriconazole, 159 to posaconazole, and 46 to isavuconazole. These ADE signals were then aggregated and sorted based on their occurrence frequency. The top 20 ADE signals were selected for analysis. For voriconazole, the highest frequency ADE was death, followed by drug interactions, hallucinations, worsening of the condition, and photosensitivity reactions. The ADE signal with the highest strength was “photochemical keratosis” (PRR = 105.868, ROR = 106.965, 95% CI: 84.915–134.740). Posaconazole’s most frequent ADE was drug interactions, followed by product use issues and hypokalemia, with the strongest signal being “antibiotic level below therapeutic” (PRR = 5053.084, ROR = 5139.216, 95% CI: 3153.396–8375.587). Isavuconazole’s most frequent ADE was death, followed by off-label use and febrile neutropenia, with the strongest signal being “hepatotoxicity” (PRR = 17.499, ROR = 17.801, 95% CI: 8.844–35.829), as shown in Table 3.

**TABLE 3:** Top 20 ADE signals by frequency.

Voriconazole				Posaconazole				Isavuconazole			
ADE	n	PRR (X^2^)	ROR (95%CI)	ADE	n	PRR (X^2^)	ROR (95%CI)	ADE	n	PRR (X^2^)	ROR (95%CI)
death	734	2.090 (436.146)	2.208 (2.046–2.383)	drug interaction	92	7.733 (535.677)	8.111 (6.574–10.008)	death	105	5.061 (354.522)	6.315 (5.073–7.859)
drug interaction	513	9.950 (4121.642)	10.604 (9.692–11.601)	product use issue	48	6.08 (341.043)	6.329 (5.063–7.912)	off label use	88	6.121 (384.208)	7.383 (5.847–9.323)
hallucination	275	11.673 (2660.468)	12.077 (10.702–13.629)	hypokalaemia	42	16.663 (690.478)	17.11 (12.837–22.803)	febrile neutropenia	12	12.137 (111.941)	12.445 (7.012–22.089)
condition aggravated	241	2.746 (2660.468)	2.804 (2.466–3.188)	drug level below therapeutic	29	138.221 (5468.931)	141.636 (103.949–192.986)	drug interaction	12	3.918 (23.397)	3.999 (2.253–7.096)
photosensitivity reaction	238	44.335 (9752.817)	45.748 (40.133–52.150)	antibiotic level below therapeutic	26	5053.084 (79310.610)	5139.216 (3153.396–8375.587)	hepatotoxicity	8	17.499 (108.529)	17.801 (8.844–35.829)
visual impairment	186	4.766 (550.795)	4.861 (4.202–5.624)	pseudoaldosteronism	25	4411.131 (65472.613)	4478.422 (2713.308–7391.812)	thrombocytopenia	8	4.165 (16.275)	4.223 (2.099–8.499)
drug level increased	171	30.240 (4712.754)	30.919 (26.529–36.035)	electrocardiogram qt prolonged	24	8.532 (158.839)	8.642 (5.821–12.830)	neutropenia	7	2.893 (6.918)	2.923 (1.385–6.169)
disease progression	145	3.578 (267.567)	3.629 (3.078–4.278)	respiratory failure	22	4.610 (64.437)	4.661 (3.115–6.975)	therapy non-responder	7	8.874 (41.409)	9 (4.265–18.993)
vision blurred	145	3.233 (222.215)	3.277 (2.779–3.863)	hepatotoxicity	20	11.263 (176.788)	11.383 (7.322–17.696)	respiratory failure	6	4.479 (12.957)	4.526 (2.022–10.131)
drug resistance	143	16.064 (1985.808)	16.355 (13.849–19.315)	drug-induced liver injury	19	9.967 (152.441)	10.072 (6.479–15.657)	disease progression	6	2.503 (4.037)	2.523 (1.127–5.648)
hallucination visual	141	21.608 (2713.417)	22.001 (18.602–26.021)	febrile neutropenia	18	4.944 (55.978)	4.988 (3.173–7.840)	visual impairment	6	2.597 (4.425)	2.619 (1.17–5.861)
respiratory failure	106	4.684 (303.415)	4.737 (3.909–5.739)	liver function test abnormal	17	7.025 (87.126)	7.089 (4.454–11.282)	multiple organ dysfunction syndrome	5	12.043 (40.208)	12.169 (5.039–29.388)
hepatic function abnormal	96	8.672 (641.149)	8.771 (7.168–10.734)	hepatocellular injury	17	18.248 (259.577)	18.420 (11.416–29.720)	nervous system disorder	5	6.226 (17.043)	6.285 (2.603–15.177)
drug level decreased	92	33.524 (2807.958)	33.926 (27.558–41.766)	pancytopenia	16	4.670 (45.491)	4.706 (2.918–7.59)	blood creatinine increased	5	4.155 (9.055)	4.191 (1.736–10.12)
hepatotoxicity	91	11.833 (885.558)	11.966 (9.723–14.726)	cardiac arrest	16	2.508 (13.080)	2.522 (1.541–4.126)	general physical health deterioration	5	2.732 (3.911)	2.752 (1.14–6.645)
treatment failure	91	3.289 (142.869)	3.317 (2.697–4.079)	treatment failure	15	2.358 (10.446)	2.370 (1.425–3.940)	underdose	4	2.842 (3.121)	2.859 (1.068–7.652)
drug-induced liver injury	87	10.001 (691.928)	10.106 (8.175–12.493)	neuropathy peripheral	13	2.029 (5.816)	2.037 (1.180–3.516)	eosinophilia	4	13.828 (35.641)	13.944 (5.209–37.329)
delirium	84	7.898 (497.272)	7.976 (6.429–9.895)	cholestasis	12	10.524 (94.035)	10.591 (6.000–18.694)	electrocardiogram qt prolonged	4	5.302 (10.006)	5.341 (1.995–14.295)
actinic keratosis	78	105.868 (7464.528)	106.965 (84.915–134.740)	hepatic enzyme increased	12	2.582 (10.125)	2.593 (1.469–4.575)	drug level increased	4	11.750 (29.335)	11.848 (4.426–31.716)
neurotoxicity	73	14.654 (906.731)	14.787 (11.730–18.642)	drug level decreased	11	17.083 (150.941)	11.383 (7.322–17.696)	drug-induced liver injury	4	7.739 (17.237)	7.802 (2.915–20.883)

### 3.3 ADE signals affecting system organ classes

**FIGURE 1:**
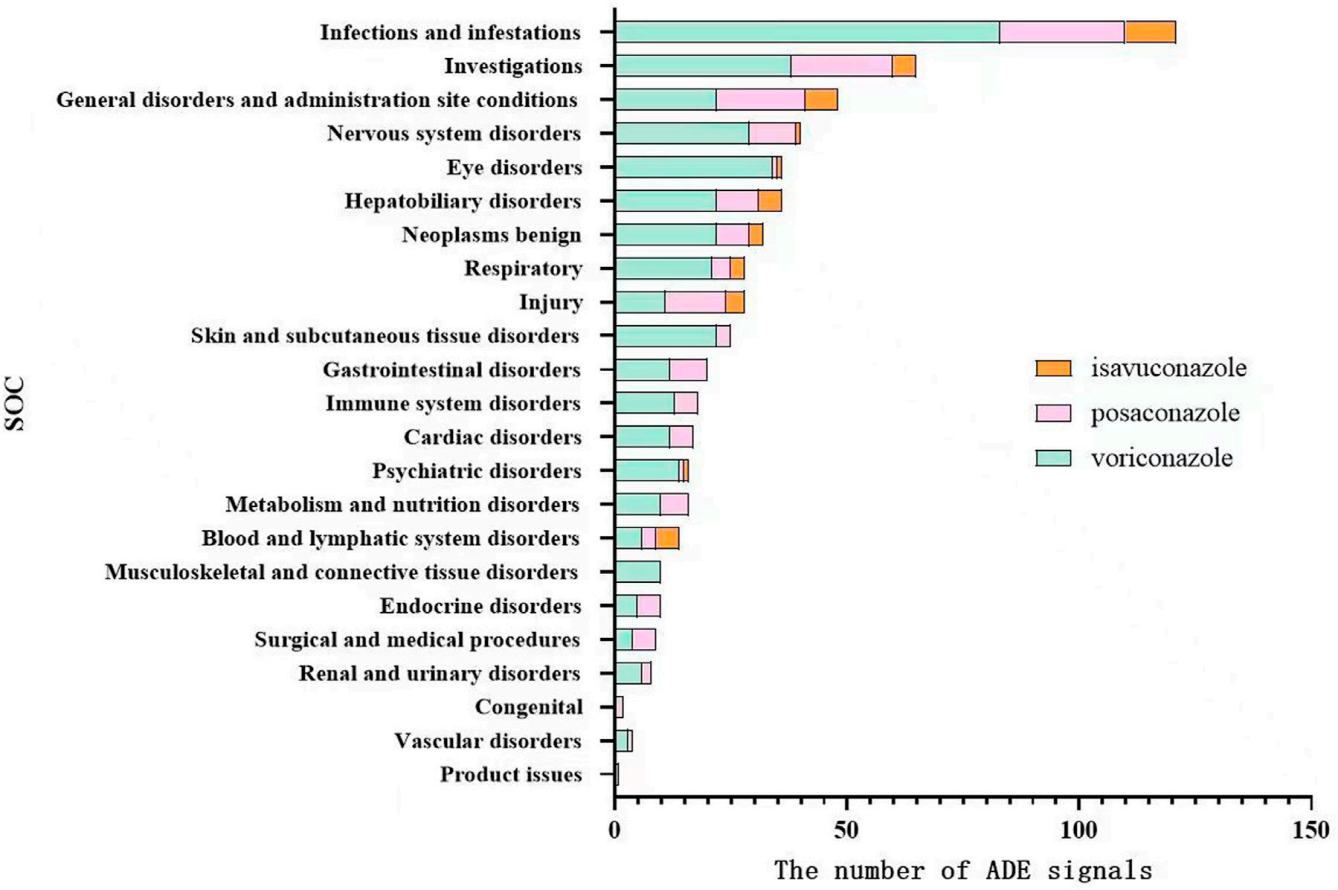
Distribution of system organ classes affected by ADE signals of voriconazole, posaconazole, and isavuconazole.

Using MedDRA 26.0, ADE signals were classified into SOCs, showing involvement in 23 SOCs overall, with voriconazole and posaconazole affecting 22 SOCs each and isavuconazole affecting 11 SOCs. ADE signals were mainly concentrated in infections and infestations, investigations, general disorders and administration site conditions, and hepatobiliary disorders. Voriconazole also had prominent ADE signals in eye disorders and nervous system disorders, as shown in Figure 1.

### 3.4 Key system organ class visualization comparison

**FIGURE 2:**
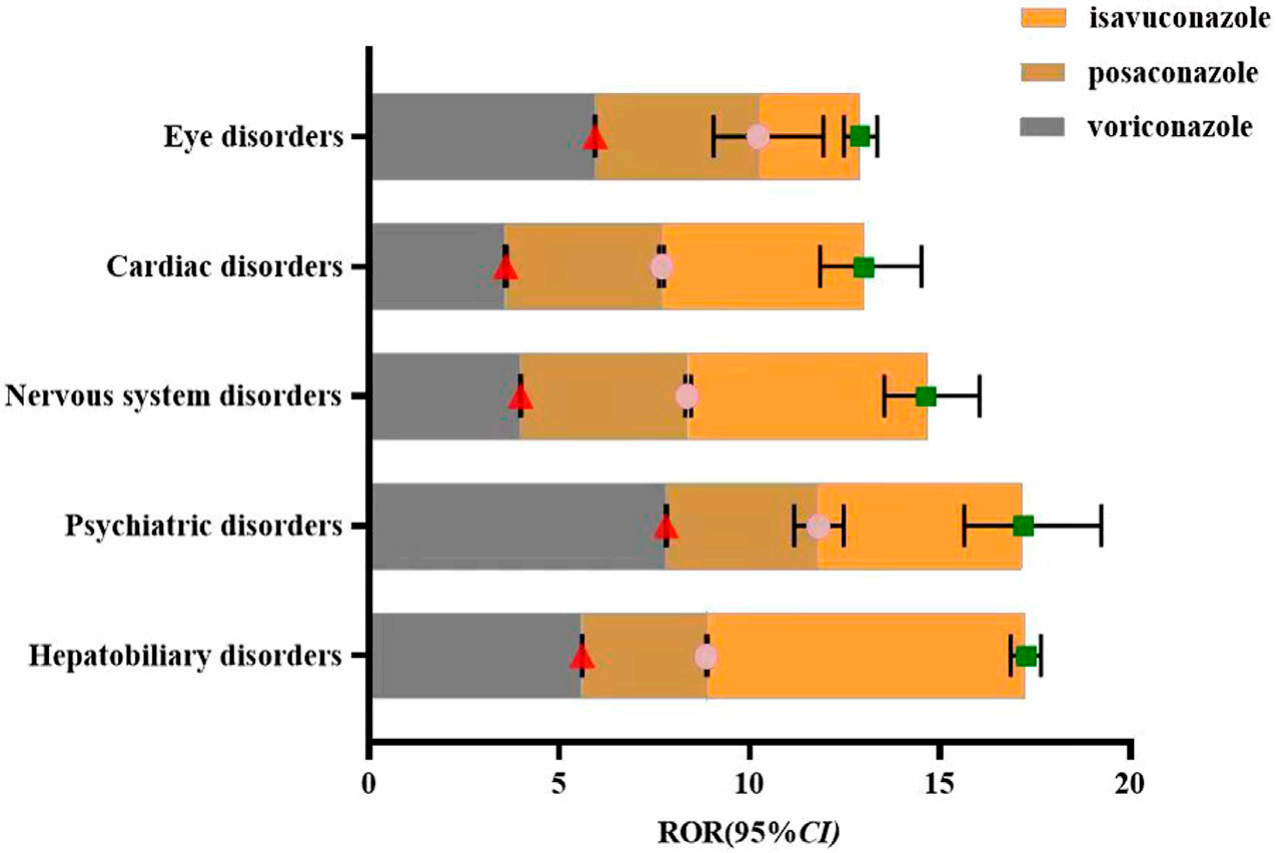
Visual comparison of key system organ classes affected by voriconazole, posaconazole, and Isavuconazole.

The common adverse reactions associated with the clinical use of voriconazole, posaconazole, and isavuconazole have been accumulated and categorized into key system organ classes. These classes will be compared visually. The ROR analysis will be conducted for the key system organ classes of all three drugs to calculate their respective ROR and 95% CI. The visualization will depict the differences in ADE signals among the key system organ classes for the three drugs, as shown in Figure 2.

## 4 Discussion

### 4.1 Comparison of ADEs among key system organ classes for three drugs

Eye disorders are common ADEs associated with voriconazole. This study detected that most eye disorder ADEs were documented in the drug’s labeling. High-frequency eye disorder ADEs include visual disturbances (186 cases), blurred vision (145 cases), photophobia (64 cases), toxic optic neuropathy (43 cases), and decreased visual acuity (36 cases). Fewer eye disorder ADEs were associated with posaconazole and isavuconazole, with only one eye disorder ADE signal detected for each drug. These were posaconazole-related visual impairment (3 cases) and isavuconazole-related visual impairment (6 cases). Some studies suggest that voriconazole’s impact on altered visual perception, color vision, and static visual field thresholds may indicate its pharmacological effects on the rod and cone pathways. It can reversibly adjust the retina to a more light-adapted state, leading to increased relative sensitivity (Zrenner et al., 2014).

Cardiac diseases: Voriconazole, posaconazole, and isavuconazole were all associated with QT interval prolongation ADEs, with 60, 25, and 4 cases respectively. According to Yap and Camm (2000), antifungal drugs may affect the IKR channel subtype of K^+^ channels, important for ventricular repolarization, or inhibit the cytochrome P450 metabolic pathway of other drugs that may also prolong the QT interval, thereby leading to QT interval prolongation. Therefore, cardiac rhythm should be closely monitored during treatment, and drug interactions should be considered, especially when co-administering potent cytochrome P450 enzyme inhibitors which may lead to cardiac-related ADEs (Panos et al., 2016). Other risk factors for QT interval prolongation include hypomagnesemia, diabetes, perioperative anesthetics, and multiple arrhythmogenic drugs. In contrast, isavuconazole ADEs included 9 cases of QT interval shortening. A study involving 26 adult patients from 7 hospitals, where patients received isavuconazole for invasive fungal infections, showed a QTc interval shortening in 24 cases, with an average decrease in QTc of 7.4% ± 5.8% (36.5 ± 38.8 ms, range 7–202; P = 0.004) during treatment (Mellinghoff et al., 2018). Tracy P et al. reported a female patient who experienced QT interval prolonga-tion with voriconazole treatment, which normalized after switching to isavuconazole (Trang et al., 2017). The QT interval shortening mechanism of isavuconazole may involve its simultaneous inhibition of HERG-mediated potassium channels and L-type calcium channels.

Psychiatric diseases: Voriconazole was particularly prominent, with hallucinations (286 cases), visual hallucinations (141 cases), and confusion (119 cases). In comparison, the other two drugs had fewer psychiatric ADE signals, with one each: hallucinations related to posaconazole (6 cases) and confusion related to isavuconazole (3 cases). The neurobiological mechanism of voriconazole-induced psychiatric symptoms is not well understood but may be related to factors such as age and immune system effects (Rekha et al., 2023). Thus, elderly patients should be closely monitored for changes in mental status when using voriconazole.

Hepatobiliary system ADEs were detected for all three drugs, with the most frequent being abnormal liver function for voriconazole (96 cases), hepatotoxicity for posaconazole (20 cases), and hepatotoxicity for isavuconazole (8 cases). Voriconazole-induced hepatotoxicity mainly involves oxidative stress, affecting various oxidative stress-related biological pathways, including cell repair, energy production, and OGlcN acylation, resulting in cellular dysfunction and alterations in energy metabolism, urea cycle, and nucleotide metabolism (Wu et al., 2020; Wu et al., 2019). Studies suggest that posaconazole hepatotoxicity may involve mitochondrial dysfunction, reducing mitochondrial membrane potential, impairing electron transport chain enzyme complex function, accumulating mitochondrial superoxide anions, decreasing mitochondrial DNA, and inducing apoptosis (Haegler et al., 2017). In contrast, isavuconazole has higher tolerability and fewer drug interactions. A phase III randomized non-inferiority trial (SECURE trial)demonstrated that isavuconazole was better tolerated than voriconazole, with lower hepatotoxicity rates (8.9% vs. 16.2%, P = 0.016), consistent with the ADE signal results from this study (Maertens et al., 2016).

### 4.2 New ADE signals

Compared to drug instructions, after excluding ADE signals related or completely unrelated to indications, this study identified new ADE signals for voriconazole: respiratory failure, rhabdomyolysis, cheilitis, bullous dermatitis, Cushing’s syndrome, and diabetes insipidus. There are two reported cases of voriconazole-induced rhabdomyolysis. Alawfi A et al. reported a 9-year-old girl who developed lower limb weakness and inability to walk during voriconazole treatment for fungal infection, confirmed by severe hypokalemia with characteristic ECG changes combined with rhabdomyolysis. The drug was discontinued, and the patient was treated with fluid and intravenous potassium, leading to clinical improvement and mobility (Alawfi et al., 2022). Li Mei et al. reported a case of voriconazole-induced rhabdomyolysis in a patient with severe hepatitis and invasive pulmonary fungal infection. The patient experienced poor appetite, generalized weakness, difficulty raising the head, and muscle pain during treatment. The patient’s myocardial enzyme spectrum was persistently elevated: creatine kinase 1541.9 U/L, creatine kinase-MB 56.1 U/L, myoglobin 4089.6 μg/mL, high-sensitivity troponin 10.26 pg/mL, with no significant abnormalities on ECG. The symptoms gradually resolved after discontinuing voriconazole (Li et al., 2017). The study suggests regular monitoring of voriconazole blood concentrations to avoid ADEs from high drug levels, and monitoring blood potassium levels, especially when using other drugs that may affect potassium levels, to identify and intervene in potential links between hypokalemia and rhabdomyolysis in time.

New ADE signals for posaconazole: respiratory failure, peripheral neuropathy, hallucinations, acute pancreatitis, and Guillain-Barré syndrome. Literature reports include a female patient who developed bilateral hand, foot, and thigh flaccid pain symptoms indicative of peripheral neuropathy during posaconazole treatment for vaginal yeast infection, which significantly improved after treatment with methylprednisolone and magnesium (Hussain et al., 2019). Another female patient developed drug-related pancreatitis symptoms on the fifth day of posaconazole treatment, which fully resolved after discontinuing posaconazole (Pilmis et al., 2017).

New ADE signals for isavuconazole: visual impairment and confusion. Dipippo et al. reported the results of long-term (≥6 months) prophylactic treatment with isavuconazole in 50 hematologic patients, showing better tolerability compared to other triazole drugs, with only 3 cases of possibly related blurred vision, which were self-limiting, and 1 case of neurotoxicity (DiPippo and Kontoyiannis, 2019).

### 4.3 Research strengths and limitations

The spontaneous reporting system for drug adverse events is essential for monitoring drug safety post-marketing, providing a comprehensive understanding of ADEs for various drugs (LaFargue et al., 2019). This study utilized real-world data and data mining techniques to obtain the distribution of ADEs for voriconazole, posaconazole, and isavuconazole across different organs, and comparatively analyzed and evaluated the differences in ADEs among the three drugs, providing a basis for rational clinical drug use. However, the study did not consider the impact of underlying diseases and concomitant medications on safety signals. Additionally, the quality and completeness of ADE reports in the FAERS database may affect the study results. Therefore, while the ROR and PRR methods used in this study calculate the association between drugs and ADEs, they do not indicate the incidence of ADEs or the biological relationship between the drugs and ADEs, necessitating further research to confirm these findings.

## 5 Conclusion

Voriconazole, posaconazole, and isavuconazole can all pose risks of hepatobiliary and cardiac organ diseases. Additionally, voriconazole has higher safety risks for ocular and nervous system diseases, while isavuconazole shows higher tolerability. For patients with posaconazole’s new ADE signal: respiratory failure, as found in this study, if respiratory failure is caused by invasive fungal infection, the patient’s respiratory improvement should be dynamically evaluated during antifungal treatment, and the effect of antifungal treatment should be evaluated in combination with relevant tests. All three drugs detected new ADE signals not mentioned in the drug instructions, particularly cases of voriconazole-induced rhabdomyolysis and posaconazole-induced peripheral neuropathy, which have been reported in the literature. These findings warrant close attention during clinical use, and further clinical studies are encouraged to evaluate the safety differences of triazole drugs.

## Data Availability

The original contributions presented in the study are included in the article/supplementary material, further inquiries can be directed to the corresponding author.
